# High-Accuracy Determination of Potassium and Selenium in Human Serum by Two-Step Isotope Dilution ICPMS

**DOI:** 10.1155/2021/5533631

**Published:** 2021-11-06

**Authors:** Jian Yang, Lixia Chi, Shengmin Li

**Affiliations:** ^1^Institute of Disaster Prevention, Langfang 065201, China; ^2^Key Laboratory of Building Collapse Mechanism and Disaster Prevention, China Earthquake Administration, Sanhe 065201, China; ^3^Beijing Institute of Medical Device Testing, Beijing Key Laboratory of Medical Device Testing and Safety Evaluation, Beijing 101111, China

## Abstract

A high-accuracy measurement technique for determining potassium and selenium in human serum was developed by using two-step Isotope Dilution Inductively Coupled Plasma-Mass Spectrometry (ICPMS) in this research. A more accessible method of the quadrupole ICPMS was employed in this research to achieve an equally high accuracy which had been achieved by a much more expensive method, namely, high-resolution sector field ICPMS (SF-ICPMS), with a comparatively easy and simple operation. In addition, we have evaluated the uncertainty of this method. The results showed that the determination limits of potassium and selenium in serum were 0.8 mg/kg and 2.7 *μ*g/kg, respectively, and the precision for both measurements was lower than 0.2% and 0.7%. The measurement, when employed to measure potassium and selenium in standard materials NIST956D, NIST909C, and GBW09152, had caused a maximum deviation of less than 0.9%, within the stated uncertainty range of standard materials. The RELA international inter-laboratory comparisons of potassium in serum in 2018 conducted by our laboratory also yielded a satisfactory result.

## 1. Introduction

Isotope Dilution ICPMS, with an edge over many conventional methods, has been recognized by CCQM as one of the top five methods with absolute measurement properties. It is also the only authoritative method to measure the values of trace elements and the ultra-trace elements [1]. Isotope Dilution ICPMS is achieved through the practice of balance weighing and the mass spectrometry measurement of isotope abundance, converting the chemical composition analysis into mass spectrometry measurement. It has absolute measurement properties, and the values obtained can be traced directly to the international basic unit of the mass, namely, mole. In conventional detection methods, factors such as the loss of tested elements during the pretreatment process of the sample, the matrix effects, and the instrument noises may affect the accuracy of the tests negatively, which can be eliminated to a great extent by the isotope dilution method [2–5]. In Isotope Dilution ICPMS, the isotope abundance ratio rather than the concentration of the sample is measured. When the isotope spike is added to the tested sample to reach a sufficient balance, the isotope abundance ratio, without the contamination by foreign isotope, will become a certain value [6–10]. In the subsequent separation and concentration processes, the change of the abundance ratio, even in case of a loss of the sample, will not be affected. Consequently, the operation procedures can be simplified and the errors caused by the preprocessing of the sample can be eliminated as well. Originated from the British Government Chemist's Laboratory (LGC) [11], the two-step Isotope Dilution ICPMS has the following advantages over traditional Isotope Dilution ICPMS methods: there is no need to calibrate the concentration of isotope spike in advance for the determination. The concentration of isotope spike can only be determined by using the expensive high-resolution sector field ICPMS (SF-ICPMS). Also, all that is needed is to get the value of a given isotope abundance ratio. The chemical components in the serum can be determined, with the requirements for the mass spectrometer consequently lowered [12–15].

In the area of medical laboratory traceability, Isotope Dilution ICPMS has also been universally acknowledged as reference method of the best accuracy for measurement. The Joint Committee for Traceability in Laboratory Medicine (JCTLM) is responsible for evaluating candidate reference materials, reference measurement procedures, and reference laboratories and will list the evaluated reference materials, reference measurement procedures, and reference measurement laboratories on the website of its secretariat. Until recently, there is no reference method for measuring serum selenium by using the Isotope Dilution ICPMS in the list published by JCTLM, with only the existence of measuring potassium in serum by Isotope Dilution ICPMS [16]. The concentrations of potassium and selenium in people's body, which are related to many diseases, are important indicators of their health condition. Potassium is the major cation to maintain the physiological activities of cells, and it is also an important electrolyte for intracellular fluid [17, 18]. The potassium ions in people's body can help maintain a dynamic balance in the exchange process between the cells and body fluids. Therefore, the determination of the concentration of potassium ions in the extracellular fluid can, to a certain extent, reflect the concentration of potassium in the cells indirectly, which helps the diagnosis of electrolytes balance or the acid-base dysequilibrium [19–21]. Selenium can help enhance immunity, and it also has the function of anti-age and anti-cancer. Deficiency or excess of selenium can cause such diseases as autoimmune thyroiditis and type 2 diabetes [22–26].

Two-step Isotope Dilution ICPMS has been established in our laboratory to achieve high-accuracy determination of potassium and selenium in human serum. The reliability of this method has been verified by its employment to the analysis of standard materials and through the RELA comparison. Up to now, there are few reports about the reference method of selenium and potassium in serum by two-step isotope dilution mass spectrometry. This method can be recommended as a traceable reference method for calibrating detection systems or assigning values to certified reference materials.

## 2. Materials

### 2.1. Materials and Reagents

The laboratory environment in this research was a class 100,000 cleanroom. The water used was provided by Water Purification System: Milli-Q Advantage (Millipore, USA). The nitric acid (Ultrapure-BVIII) and hydrogen peroxide (30% H_2_O_2_, Ultrapure-BVIII) were produced by Beijing Institute of Chemical Reagents, China. The microanalytical balance used for sample weighing in this experiment was Mettler Toledo XS205 (Switzerland); the microwave digestion instrument for sample preparation was MARS (Pynn, USA), and the sample analysis was conducted in ICP mass spectrometer: Elan DRC-e (PerkinElmer, USA). The standard materials used for method traceability were selenium (Se) standard solution SRM3149 (NIST USA), potassium (K) standard solution SRM3141a (NIST, USA); isotope spikes used in the method were ^41^K (Assay: 95%, ISOFLEX, USA) and ^78^Se (Assay: 98.8%, Oak Ridge, USA). The accuracy verification was achieved by selenium in human serum SRM909c (NIST, USA), electrolytes in frozen human serum SRM 956d (NIST, USA), and inorganic components in frozen human serum GBW09152 (National Institute of Metrology, China).

### 2.2. Instrument Parameters

The instrument parameters for this research are listed in Table 1.

## 3. Methods

### 3.1. Mass Spectrometry Procedures

The concentrations of potassium and selenium in the serum sample were calculated according to concentration formula (1) in the two-step Isotope Dilution ICPMS [27].

(1) $$\begin{array}{c} Cx=C_{z}\cdot \frac{m_{Z_{c}}}{m_{Y_{c}}}\cdot \frac{m_{Y}}{m_{X}}\cdot \frac{R_{Y}-\kappa \cdot R_{B}}{\kappa \cdot R_{B}-R_{Z}}\cdot \frac{R_{Z}-R_{B_{c}}}{R_{B_{c}}-R_{Y}}\;-C_{B}\;, \end{array}$$

where *C*_z_ is the concentration of the standard solution. In the mixture of the serum sample and the enriched isotope, *m*_Y_ is the mass of the enriched isotope, while *m*_X_ is the mass of the serum sample. In the mixture of the standard solution and the enriched isotope, m_Yc_ is the mass of the enriched isotope, while m_zc_ is the mass of the standard solution. *R*_*z*_ is the isotope abundance ratio of ^39^ K/^41^K or ^80^Se/^78^Se in the standard solution. *R*_Y_ is the abundance ratio of ^39^ K/^41^K or ^80^Se/^78^Se in the enriched isotope. *R*_B_ is the abundance ratio of ^39^ K/^41^K or ^80^Se/^78^Se in the mixture of the serum sample and the enriched isotope. *R*_Bc_ is the abundance ratio of ^39^ K/^41^K or^80^Se/^78^Se in the mixture of the standard solution and the enriched isotope. *k* is the correction coefficient of the reference sample and the serum sample. *C*_B_ is the blank of the measurement process.

In order to obtain the best accuracy in the isotope ratio measurement, an effort should be made to make *R*_B_ ≈ *R*_Bc_≈1 [28]. In the experiment, it had been found that the fluctuation of *R*_z_ and *R*_Y_ barely had effects on the final results, whereas the fluctuation of *R*_B_ and R_Bc_ did affect the final results. Therefore, *R*_B_ and R_Bc_ had to be measured alternately, and at the same time, the error caused by the measurement drift of the instrument could be eliminated. Each mixture of the serum sample containing selenium and the enriched isotope had been measured alternately 3 times, and each mixture of the serum sample containing potassium and the enriched isotope had been measured alternately 6 times.

### 3.2. Preparation of the Solution

The weighing method was used to perform a secondary dilution for NIST SRM3141a at a ratio of approximately 1 : 400 and a tertiary dilution for NIST SRM3149 at a ratio of approximately 1 : 8000. The final dilution must be made on the exact day of the experiment.

Enriched isotopes ^41^K as KCL and ^78^Se selenium powder were separately dissolved by using BVIII grade nitric acid and then diluted with ultrapure water to an appropriate concentration. A secondary dilution was carried out on the exact day of the experiment.

The weighing method was used to make the mixed solution of SRM3141a and ^41^K spike solution and the mixed solution of serum sample and ^41^K spike solution. An effort was made to guarantee the isotope ratio of the two mixed solutions (^39^ K/^41^K) close to 1, and at the same time, the values of the cps signal intensity of 39K and 41K in the two mixed solutions were close.

1 g～1.3 g of serum was accurately weighed and put in a microwave digestion tank, with an appropriate amount of ^78^Se spike solution added. We made sure that *m*_Y_ of the added ^78^Se spike solution was accurately weighed to make the value of ^80^Se/^78^Se close to 1 so as to achieve a better accuracy. After 10 ml of HNO_3_+1mLH_2_O_2_ was added, the digestion tank was digested in a microwave digestion instrument by way of gradient heating with the maximum temperature of the digestion being 180°C for a time span of 40 minutes. After the microwave digestion stood still overnight after the digestion, it was moved to ISOLSB PP beaker at 150°C and steamed until the solution volume was reduced to about 2 ml, and then H_2_O_2_ (10%) was added to drive the acid repeatedly 6 times until the sample solution was colorless. Then, a value of 30 ml was set and its isotope ratio was measured on the exact day of the experiment. A mixed solution of isotope spikes of SRM3149 and ^78^Se was made to make sure the value of ^80^Se/^78^Se was close to 1, and at the same time, the values of the cps signal intensity of 80Se and 78Se in the two mixed solutions were close.

On the exact day of the experiment, a potassium solution and a selenium solution with appropriate concentrations were separately made to make the signal intensity of ^39^K (^80^Se) in the solutions consistent with that of the mixed solution mentioned previously.

On the exact day of the experiment, a ^41^K solution and a ^78^Se solution with appropriate concentrations were prepared separately to make the signal intensity of ^41^K (^78^Se) in the solutions consistent with that of the mixed solution mentioned previously.

## 4. Results and Discussion

### 4.1. The Selection of Methane Reagent Gas Flow

In this experiment, the dynamic reaction cell of ICPMS was used to eliminate the interference. When selenium was measured, there was a serious interference of Ar_2_ due to the fact that the interference of Ar_2_ to ^80^Se is more serious than its interference to ^78^Se [29]. Therefore, an appropriate flow rate of the reactant gas CH_4_ and argon should be selected so as to obtain the most reliable results for the measurement of ^80^Se. In this research, the most appropriate flow rate of the reactant gas CH_4_, namely, 0.8 mL/min of methane and 0.45 mL/min of argon, was selected by using the dynamic reaction cell of ICPMS. When potassium was measured, there was a serious interference of ArH, CaH, and MgO, especially to ^41^K. Therefore, an appropriate flow rate of the reactant gas CH_4_ and argon should be selected so as to obtain the most reliable results for the measurement of ^41^K. The most appropriate flow rate of the reactant gas CH_4_, namely, 1.5 mL/min of methane and 0.8 mL/min of argon, was selected by using the dynamic reaction cell of ICPMS.

### 4.2. The Impact of the Concentration of Nitric Acid in the Solution on the Value of ^80^Se/^78^Se

**Figure 1:**
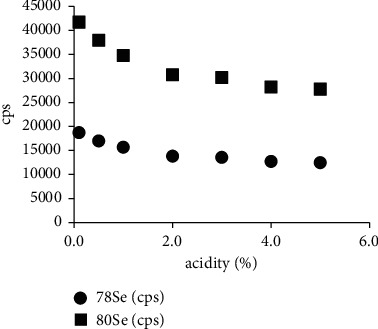
The response signal of ^78^Se and ^80^Se with acidity.

**Figure 2:**
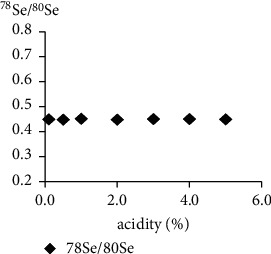
The value of ^78^Se and ^80^Se with acidity.

Solutions with different concentrations of nitric acid and the same amount of Se (5.0 *μ*g/L) were made and then a measurement of the signal intensities (cps) of ^78^Se and ^80^Se was conducted under the same experimental conditions. The results showed that the cps response signal of ^78^Se and ^80^Se decreased with the increase of acidity (Figure 1), but the value of ^80^Se/^78^Se hardly changed (Figure 2). Therefore, it can be concluded that the difference in acidity can be ignored when preparing the reference solution and the sample solution, but the analysis sensitivity is higher when the acidity is relatively low.

### 4.3. Interference Verification and the Calculation of Correction Coefficient

The signal intensities of ^39^K, ^41^ K, ^80^Se, and ^78^Se in ultrapure water have been tested under the established experimental conditions. The results show that the ratio of the signal response intensity of ultrapure water to the signal intensity of the corresponding isotope in the serum sample is less than 0.1%. Therefore, it is safely believed that the interference of Ar_2_ or ArH has been eliminated by the way of dynamic reaction cell, or to be more specific, their interference on ultrapure water can be ignored. Then, the ratio of the isotope abundance ratio (*R*_*Z*_) of ^39^ K/^41^K (^80^Se/^78^Se) in the standard solution to the isotope abundance ratio (*R*_*S*_) of ^39^ K/^41^K (^80^Se/^78^Se) in the serum sample was tested. In the case that the ratio *R*_*Z*_ to *R*_*S*_ ranges from (100.0–0.1) % to (100.0 + 0.1) %, it can be concluded that the matrix interference of serum samples can be ignored, for the measurement precision of the instrument is of the same error level. In this experiment, ^39^ K/^41^K in the potassium standard solution is consistent with ^39^ K/^41^K in the serum sample, while ^80^Se/^78^Se in the selenium standard solution is greater than ^80^Se/^78^Se in the serum sample. After the microwave digestion of the serum selenium sample, the interference of the serum matrix on ^80^Se/^78^Se ratio has been reduced; nevertheless, it has been proved that the ratios of ^80^Se/^78^Se at different concentrations levels are consistent. According to calculation formula (2) of the correction coefficient, the correction coefficient of potassium was 1 and that of selenium was about 1.027, and we made sure that the correction coefficient of selenium was determined simultaneously on the exact day of the experiment.

(2) $$\begin{array}{c} k=\frac{R_{Z}}{R_{S}}. \end{array}$$

### 4.4. The Process Blanks (LoB) and Detection Limits (LoD)

While preparing the mixed solution of serum sample and enriched isotope, we added an appropriate amount of ^78^Se (or ^41^K) enriched isotope into the blank sample tube as the process blank, where the value of ^80^Se/^78^Se (or ^39^ K/^41^K) was about 2. The process blank, together with the sample, went through the entire preprocessing procedure. In accordance with the requirements of International Union of Pure and Applied Chemistry (IUPAC), the sample blank should be tested 20 times repeatedly according to the formula LoD = LoB + *k*^*∗*^*S*_b1_ (*k* = 2, and *S*_b1_ is the standard deviation of the blank sample). The results showed that the process blanks of potassium and selenium in serum were −0.3 mg/kg～0.3 mg/kg and 1.0 *μ*g/kg～1.50 *μ*g/kg, respectively, and when the confidence interval of 95% was determined, the detection limits of potassium and selenium in serum were 0.8 mg/kg and 2.7 *μ*g/kg, respectively.

### 4.5. The Precision of the Method

Assigned value experiments were conducted on the candidate standard materials of potassium and the candidate standard materials of selenium in human serum in our laboratory. The experiments were carried out in two days, with 6 bottles per day. The results are shown in Table 2. The measurement precision for potassium and selenium in serum was lower than 0.2% and 0.7%, respectively.

### 4.6. The Trueness of the Method

The measurement was employed to determine potassium and selenium in standard materials NIST956D, NIST909C, and GBW09152. The results are shown in Table 3, where the maximum deviation was less than 0.9%, within the stated uncertainty range of standard materials.

### 4.7. Result of RELA 2018

**Figure 3:**
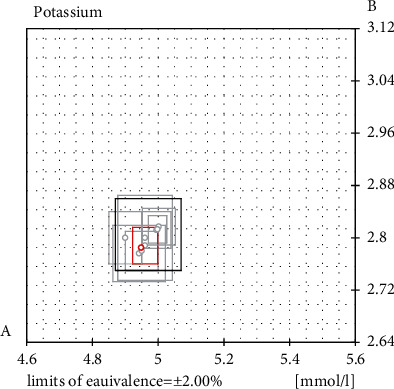
Result of RELA 2018.

Our laboratory had joined the RELA international inter-laboratory comparisons of potassium in serum in 2018. In Figure 3 and Table 4, lab code 115 showed the results obtained by our laboratory. The result is in the center of the acceptable data range. We evaluated the combined standard uncertainty of the results of RELA. The uncertainty caused by the sample weighing, the balance itself, the single measurement, and the uncertainty caused by the vial to vial difference, the traceability standard material itself, and by the process blanks are all taken into consideration in the process of evaluation. The combined standard uncertainty for potassium in sample A and sample B was 0.04 mmol/L and 0.03 mmol/L, respectively (*k* = 2).

### 4.8. Evaluation of Uncertainty in Measurements

In this research, the uncertainty caused by factors such as the experimental reagents, the samples, the laboratory environments, the solution preparation, the instrument measurement, and the data processing has been evaluated as the source of uncertainty in the measurement process. It can be concluded that by evaluating the uncertainty of each parameter in formula (1), the uncertainty caused by each factor in the measurement process can be fully included. Each parameter in formula (1) is an independent parameter, and the uncertainty  *u*_*c*_(*y*) related to measurement is calculated as follows:

(3) $$\begin{array}{c} u_{c}\left(y\right)=\sqrt{\overset{N}{\underset{i=1}{\sum }}{\left[\frac{\partial f}{\partial x_{i}}\right]}^{2}u^{2}\left(x_{i}\right)}. \end{array}$$

The formula of the sensitivity coefficient ((∂f/∂x_i_)) of each parameter in formula (1) is as follows:

(4) $$\begin{array}{c} \frac{\partial {\text{C}}_{\text{x}}}{\partial {\text{C}}_{\text{z}}}=\frac{m_{Z_{c}}}{m_{Y_{c}}}\cdot \frac{m_{Y}}{m_{X}}\cdot \frac{R_{Y}-K\cdot R_{B}}{K\cdot R_{B}-R_{Z}}\cdot \frac{R_{Z}-R_{B_{c}}}{R_{B_{c}}-R_{Y}},\\ \frac{\partial {\text{C}}_{\text{x}}}{\partial {\text{m}}_{\text{Y}}}=C_{z}\cdot \frac{m_{Z_{c}}}{m_{Y_{c}}}\cdot \frac{1}{m_{X}}\cdot \frac{R_{Y}-K\cdot R_{B}}{K\cdot R_{B}-R_{Z}}\cdot \frac{R_{Z}-R_{B_{c}}}{R_{B_{c}}-R_{Y}},\\ \frac{\partial {\text{C}}_{\text{x}}}{\partial {\text{m}}_{{\text{Y}}_{\text{c}}}}=C_{z}\cdot \frac{-m_{Z_{c}}}{m_{Y_{c}}^{2}}\cdot \frac{m_{Y}}{m_{X}}\cdot \frac{R_{Y}-K\cdot R_{B}}{K\cdot R_{B}-R_{Z}}\cdot \frac{R_{Z}-R_{B_{c}}}{R_{B_{c}}-R_{Y}},\\ \frac{\partial {\text{C}}_{\text{x}}}{\partial {\text{m}}_{\text{x}}}=C_{z}\cdot \frac{m_{Z_{c}}}{m_{Y_{c}}}\cdot \frac{-m_{Y}}{m_{X}^{2}}\cdot \frac{R_{Y}-K\cdot R_{B}}{K\cdot R_{B}-R_{Z}}\cdot \frac{R_{Z}-R_{B_{c}}}{R_{B_{c}}-R_{Y}},\\ \frac{\partial {\text{C}}_{\text{x}}}{\partial {\text{m}}_{{\text{Z}}_{\text{c}}}}=C_{z}\cdot \frac{1}{m_{Y_{c}}}\cdot \frac{m_{Y}}{m_{X}}\cdot \frac{R_{Y}-K\cdot R_{B}}{K\cdot R_{B}-R_{Z}}\cdot \frac{R_{Z}-R_{B_{c}}}{R_{B_{c}}-R_{Y}},\\ \frac{\partial {\text{C}}_{\text{x}}}{\partial {\text{R}}_{\text{z}}}=C_{z}\cdot \frac{m_{Z_{c}}}{m_{Y_{c}}}\cdot \frac{m_{Y}}{m_{X}}\cdot \left[\frac{R_{Y}-K\cdot R_{B}}{{\left(K\cdot R_{B}-R_{Z}\right)}^{2}}\frac{R_{Z}-R_{B_{c}}}{R_{B_{c}}-R_{Y}}+\frac{R_{Y}-K\cdot R_{B}}{K\cdot R_{B}-R_{Z}}\cdot \frac{1}{R_{B_{c}}-R_{Y}}\right],\\ \frac{\partial {\text{C}}_{\text{x}}}{\partial {\text{R}}_{\text{Y}}}=C_{z}\cdot \frac{m_{Z_{c}}}{m_{Y_{c}}}\cdot \frac{m_{Y}}{m_{X}}\cdot \frac{R_{Z}-R_{B_{c}}}{K\cdot R_{B}-R_{Z}}\cdot \frac{\left(R_{B_{c}}-R_{Y}\right)+\left(R_{Y}-K\cdot R_{B}\right)}{{\left(R_{B_{c}}-R_{Y}\right)}^{2}},\\ \frac{\partial {\text{C}}_{\text{x}}}{\partial {\text{R}}_{\text{B}}}=C_{z}\cdot \frac{m_{Z_{c}}}{m_{Y_{c}}}\cdot \frac{m_{Y}}{m_{X}}\cdot \frac{R_{Z}-R_{B_{c}}}{R_{B_{c}}-R_{Y}}\cdot \frac{-K\left(K\cdot R_{B}-R_{Z}\right)-K\left(R_{Y}-K\cdot R_{B}\right)}{{\left(K\cdot R_{B}-R_{Z}\right)}^{2}},\\ \frac{\partial {\text{C}}_{\text{x}}}{\partial R_{B_{c}}}=C_{z}\cdot \frac{m_{Z_{c}}}{m_{Y_{c}}}\cdot \frac{m_{Y}}{m_{X}}\cdot \frac{R_{Y}-K\cdot R_{B}}{K\cdot R_{B}-R_{Z}}\cdot \frac{-\left(R_{B_{c}}-R_{Y}\right)-\left(R_{Z}-R_{B_{c}}\right)}{{\left(R_{B_{c}}-R_{Y}\right)}^{2}},\\ \frac{\partial {\text{C}}_{\text{x}}}{\partial K}=C_{z}\cdot \frac{m_{Z_{c}}}{m_{Y_{c}}}\cdot \frac{m_{Y}}{m_{X}}\cdot \frac{R_{Z}-R_{B_{c}}}{R_{B_{c}}-R_{Y}}\cdot \frac{-R_{B}\left(K\cdot R_{B}-R_{Z}\right)-R_{B}\left(R_{Y}-K\cdot R_{B}\right)}{{\left(K\cdot R_{B}-R_{Z}\right)}^{2}},\\ \frac{\partial {\text{C}}_{\text{x}}}{\partial C_{B}}=-1. \end{array}$$

Therefore,

(5) $$\begin{array}{c} u_{c}\left(y\right)=\sqrt{{\left(\frac{\partial C_{x}}{\partial C_{z}}\right)}^{2}\cdot {\left(u_{c}\left(Cz\right)\right)}^{2}+{\left(\frac{\partial C_{x}}{\partial m_{Y}}\right)}^{2}\cdot {\left(u_{c}\left(m_{Y}\right)\right)}^{2}+\ldots +{\left(\frac{\partial C_{x}}{\partial C_{B}}\right)}^{2}\cdot {\left(u_{c}\left(C_{B}\right)\right)}^{2}}. \end{array}$$

This research has evaluated the uncertainty of measurement for the data in Table 2.  Table 5 shows the source of uncertainty for measuring potassium in serum, and Table 6 shows the source of uncertainty for measuring the parameters of selenium in serum. The uncertainty of the measurement of type A is the experimental standard deviation of the 6 repeated instrument measurements, taking the worst result in the experiment as the evaluation data. The uncertainty of the measurement of type B is the uncertainty of solution preparation, which is synthesized from the uncertainty resulting from the electronic balance calibration and the uncertainty resulting from weighing.

## 5. Conclusions

The two-step Isotope Dilution ICPMS established in our laboratory measured accurately potassium and selenium in human serum. The results show that this method, which is easy to operate, has a high accuracy and good repeatability. Since the concentration of potassium in serum is of ppm level, the sample was diluted more than 100 times, leaving the matrix effect on serum almost neglected, and thus there was no need for complicated preprocessing for the sample. At the same time, the concentration of selenium in serum is of ppb level. Thus, another sample was then diluted at a lower ratio, leaving an obvious serum matrix effect. In addition, this sample was microwave digested for the elimination of the matrix interference. There is no need to measure the exact concentration of the enriched isotope in the two-step isotope dilution mass spectrometry process, lowering the requirements on the mass spectrometry equipment [30, 31]. This method has been proved to be a reliable reference method for assigning values of standard materials. The values obtained can be traced directly to international system of units (SI).

## Figures and Tables

**Table 1:** Instrument parameters.

Parameter	*K*	Se
ICP RF power (W)	1100	1100
Gas flows (L/min)	0.97	0.97
Lens voltage (V)	6.75	6.75
Analog stage voltage (V)	−1700	−1700
Pulse stage voltage (V)	900	900
Dwell time per AMU	2ms	2ms
Scan mode	Peak hopping	Peak hopping
Sweeps/reading	25	25
Readings/replicate	20	25
Replicates	15	25
Cell gas A : CH_4_ (mL/min)	1.5	0.8
RP q: Ar (mL/min)	0.8	0.45

**Table 2:** Precision for potassium and selenium in human serum by two-step ID-ICPMS.

	K (mg kg^−1^)						Se (*μ*g kg^−1^)					
	Level 1		Level 2		Level 3		Level 1		Level 2		Level 3	
	Day 1	Day 2	Day 1	Day 2	Day 1	Day 2	Day 1	Day 2	Day 1	Day 2	Day 1	Day 2
1#	137.2	137.1	178.2	177.3	252.0	252.3	80.3	81.3	115.7	115.8	135.1	133.6
2#	136.6	136.9	177.7	177.6	252.7	252.0	79.6	80.8	116.6	116.1	133.2	133.9
3#	136.1	136.7	178.1	178.1	253.0	252.3	80.7	80.8	115.7	116.1	134.5	133.6
4#	136.9	137.0	178.0	178.8	252.9	252.0	80.5	80.0	114.7	116.4	132.6	134.8
5#	136.4	137.0	177.9	178.0	252.0	252.0	81.2	81.1	115.1	115.9	131.8	134.1
6#	136.9	136.9	177.9	178.6	252.3	252.4	80.7	80.5	116.6	116.2	133.6	134.5
Avg	136.8		178.0		252.3		80.6		115.9		133.8	
s	0.31		0.41		0.36		0.49		0.57		0.94	
CV	0.2%		0.2%		0.1%		0.6%		0.5%		0.7%	
U (*k* = 2)	0.14		0.21		0.23		1.5		2		2.6	

**Table 3:** Analysis of standard reference material by two-way ID-ICPMS.

	K (mg kg^−1^)				Se (*μ*g kg^−1^)	
	NIST956D			GBW09152	NIST909C	GBW09152
	Level 1	Level 2	Level 3			
Test 1	218.43	140.78	62.52	166.7	116.3	76.3
Test 2	219.66	141.21	62.39	168.0	115.9	76.5
Test 3	219.74	141.69	61.04	167.6	115.8	75.2
Avg	219.28	141.23	61.98	167.43	116.00	76.00
Certified values	219.87 ± 1.86	142.47 ± 1.37	61.50 ± 0.53	168.3 ± 3.4	115.9 ± 3.2	75.7 ± 1.6
Bias (%)	0.3%	0.9%	−0.8%	0.5%	−0.1%	−0.4%

**Table 4:** The data obtained from RELA 2018.

Lab code	A	e.u.A	B	e.u.B	Method
3	4.942	0.08	2.776	0.043	ICP-OES
24	4.9	0.05	2.8	0.04	FES
27	5.002	0.05	2.817	0.028	ICP-ID/SFMS
39	4.96	0.083	2.8	0.065	FES
51	4.96	0.038	2.788	0.028	ICPMS
54	4.995	0.044	2.812	0.028	ICPMS
63	4.95	0.05	2.78	0.03	FAAS
87	4.998	0.028	2.813	0.021	Ion chromatography
115	4.948	0.04	2.785	0.03	ID/ICPMS

**Table 5:** Sources of uncertainty in the determination of K.

Sources of uncertainty	K					
	Level 1		Level 2		Level 3	
	Value (*x*_*i*_)	*u*_*c*_ (*x*_*i*_)	Value (*x*_*i*_)	*u*_*c*_ (*x*_*i*_)	Value (*x*_*i*_)	*u*_*c*_ (*x*_*i*_)
*Type A uncertainties*						
*R*_Y_	0.041	0.00017	0.041	0.00017	0.041	0.00017
*R*_z_	11	0.063	11	0.063	11	0.063
R_Bc_	0.97	0.0014	1.0	0.0015	1.1	0.0018
*R*_B_	0.93	0.0013	1.0	0.0015	0.91	0.0014
*C*_B_/mg kg^−1^	0.14	0.16	0.16	0.16	0.21	0.16
K	—	—	—	—	—	—

*Type B uncertainties*						
*C*_z_/mg kg^−1^	46	0.10	46	0.10	46	0.10
m_zc_/mg	3.3 × 10^3^	0.040	3.3 × 10^3^	0.040	3.3 × 10^3^	0.040
m_Yc_/mg	3.4 × 10^3^	0.040	3.4 × 10^3^	0.040	3.4 × 10^3^	0.040
*m*_Y_/mg	3.4 × 10^3^	0.040	3.4 × 10^3^	0.040	3.4 × 10^3^	0.040
*m*_x_/mg	2.1 × 10^3^	0.040	1.1 × 10^3^	0.040	8.0 × 10^2^	0.040
Combined type A and type B	—	0.22	—	0.34	—	0.37
Degrees of freedom (*V*_eff_)		11		11		11
Coverage factor (k)	—	2	—	2	—	2
Measured value/mg kg^−1^	136.8	—	178.0	—	252.3	—
Expanded uncertainty (U ($\bar{x}$))	—	0.14	—	0.21	—	0.23

**Table 6:** Sources of uncertainty in the determination of Se.

Sources of uncertainty	Se					
	Level 1		Level 2		Level 3	
	Value (*x*_*i*_)	*u*_*c*_ (*x*_*i*_)	Value (*x*_*i*_)	*u*_*c*_ (*x*_*i*_)	Value (*x*_*i*_)	*u*_*c*_ (*x*_*i*_)
*Type A uncertainties*						
*R*_Y_	0.020	0.0011	0.021	0.0013	0.024	0.0018
*R*_z_	2.2	0.010	2.2	0.011	2.1	0.011
R_Bc_	1.1	0.0053	1.0	0.0032	0.96	0.0042
*R*_B_	0.98	0.011	1.1	0.011	0.89	0.011
*C*_B_/	1.4	0.37	1.4	0.37	1.4	0.37
K	1.0	0.0068	1.0	0.0068	1.0	0.0068

*Type B uncertainties*						
Cz/*μ*g kg^−1^	5.6 × 10^2^	3.0	5.0 × 10^2^	2.6	5.2 × 10^2^	2.8
m_zc_/mg	7.1 × 10^2^	0.040	7.0 × 10^2^	0.040	7.5 × 10^2^	0.040
m_Yc_/mg	2.9 × 10^2^	0.040	2.9 × 10^2^	0.040	2.5 × 10^2^	0.040
*m*_Y_/mg	1.0 × 10^2^	0.040	1.0 × 10^2^	0.040	1.1 × 10^2^	0.040
*m*_x_/mg	1.2 × 10^3^	0.040	1.0 × 10^3^	0.040	9.0 × 10^2^	0.040
Combined type A and type B	—	2.5	—	3.4	—	4.2
Degrees of freedom (*V*_eff_)		5		5		5
Coverage factor (k)		2	—	2	—	2
Measured value/mg kg^−1^	80.6	—	115.9	—	133.8	—
Expanded uncertainty (U ($\bar{x}$))	1.5		2.0		2.6	

## Data Availability

The data used to support the findings of this study are included within the article.
